# Soluble TNF-like weak inducer of apoptosis (TWEAK) enhances poly(I:C)-induced RIPK1-mediated necroptosis

**DOI:** 10.1038/s41419-018-1137-1

**Published:** 2018-10-22

**Authors:** Mohamed A. Anany, Jennifer Kreckel, Simone Füllsack, Alevtina Rosenthal, Christoph Otto, Daniela Siegmund, Harald Wajant

**Affiliations:** 1 Division of Molecular Internal Medicine, Department of Internal Medicine II, University Hospital Würzburg, Röntenring 11, 97070 Würzburg, Germany; 2 Division of Genetic Engineering and Biotechnology, Department of Microbial Biotechnology, National Research Centre, El Buhouth Street, Dokki, 12622 Giza, Egypt; 3 Experimental Surgery, Department of General, Visceral, Vascular, and Pediatric Surgery, University Hospital of Würzburg, Oberdürrbacher Strasse 6, 97080 Würzburg, Germany

## Abstract

TNF-like weak inducer of apoptosis (TWEAK) and inhibition of protein synthesis with cycloheximide (CHX) sensitize for poly(I:C)-induced cell death. Notably, although CHX preferentially enhanced poly(I:C)-induced apoptosis, TWEAK enhanced primarily poly(I:C)-induced necroptosis. Both sensitizers of poly(I:C)-induced cell death, however, showed no major effect on proinflammatory poly(I:C) signaling. Analysis of a panel of HeLa-RIPK3 variants lacking TRADD, RIPK1, FADD, or caspase-8 expression revealed furthermore similarities and differences in the way how poly(I:C)/TWEAK, TNF, and TRAIL utilize these molecules for signaling. RIPK1 turned out to be essential for poly(I:C)/TWEAK-induced caspase-8-mediated apoptosis but was dispensable for this response in TNF and TRAIL signaling. TRADD-RIPK1-double deficiency differentially affected poly(I:C)-triggered gene induction but abrogated gene induction by TNF completely. FADD deficiency abrogated TRAIL- but not TNF- and poly(I:C)-induced necroptosis, whereas TRADD elicited protective activity against all three death inducers. A general protective activity against poly(I:C)-, TRAIL-, and TNF-induced cell death was also observed in FLIP_L_ and FLIP_S_ transfectrants.

## Introduction

Tumor necrosis factor (TNF)-like weak inducer of apoptosis (TWEAK) is a member of the TNF superfamily (TNFSF). TWEAK exerts its biological activities by stimulation of fibroblast growth factor-inducible-14 (Fn14), which is a TRAF2-interacting receptor of the TNF receptor superfamily (TNFRSF)^1^. The TWEAK/Fn14 system induces pleiotropic cellular activities ranging from proinflammatory gene induction over stimulation of angiogenesis, proliferation, and cellular differentiation to cell migration and, in rare cases, apoptosis induction. Studies with Fn14 and TWEAK knockout mice implicated the TWEAK/Fn14 system in tissue repair after muscle injury and in tissue regeneration after pancreatectomy and hepatectomy^2–4^. Nevertheless, the majority of studies recognized the TWEAK/Fn14 system as a crucial factor that promotes adverse effects, e.g., fibrosis and inflammation, in situations of overshooting or chronic regenerative responses. Accordingly, blockade or deficiency of Fn14 (or TWEAK) elicited favorable therapeutic effects in a variety of disease models caused by quite different insults reaching from autoimmunity over cancer to infection and mechanical damage^1^. TWEAK obtained its name due to its ability to trigger apoptosis in a small subset of cell lines^5^. This is somewhat surprising, because Fn14 has no death domain characterizing the prototypic death-inducing receptors of the TNFRSF, such as TNFR1 and CD95. The unexpected name giving apoptosis-inducing activity of the TWEAK/Fn14 system has been traced back to a cooperative indirect mechanism comprising (i) sensitization for death receptor-induced killing by depletion of protective TRAF2-cIAP1 and TRAF2-cIAP2 complexes, and (ii) cell-type-specific induction of TNF and subsequent stimulation of the prototypic death receptor TNFR1^6–8^. It is worth mentioning that depletion of TRAF2-cIAP1/2 complexes also enables TWEAK to dampen the proinflammatory responses of TNFR1 and other TRAF2 utilizing TNFRSF receptors, e.g., CD40^9,10^. Interferon-γ-activated monocytes and macrophages are the major sources of TWEAK^11–13^ but are also prominent producers of TNF. The co-occurrence of TWEAK and TNF suggests that TNFR1-Fn14 cooperation has broad relevance in vivo. It is noteworthy that pathogen- and damage-associated molecular pattern (PAMP/DAMP)-sensing receptors and receptors of the TNFRSF, especially TNFR1, utilize an overlapping set of signaling molecules, including caspase-8, TRAF family members, and the death domain proteins TRADD, FADD, and RIPK1^14–16^.

In view of the well-established cooperativity of TWEAK/Fn14 and TNFR1 signaling, we investigated therefore here the possible crosstalk of Fn14 and polyinosinic:polycytidylic acid (poly(I:C)), a synthetic analog of double-stranded RNA, which stimulates the membranous PAMP receptor Toll-like receptor 3 (TLR3) and the cytosolic PAMP sensors retinoic acid inducible gene I and melanoma differentiation-associated protein 5^17,18^. We found that TWEAK enhances poly(I:C)-induced apoptosis and necroptosis independent from TNF induction. Our studies revealed furthermore that FLIP_L/S,_ TRADD, RIPK1, FADD, and caspase-8 have common but also non-overlapping functions in poly(I:C)-, TNF-, and TNF-related apoptosis-inducing ligand (TRAIL)-induced signaling.

## Results

### Soluble TWEAK and cycloheximide sensitize HeLa-RIPK3 and HaCaT cells for poly(I:C)-induced cell death

**Fig. 1:**
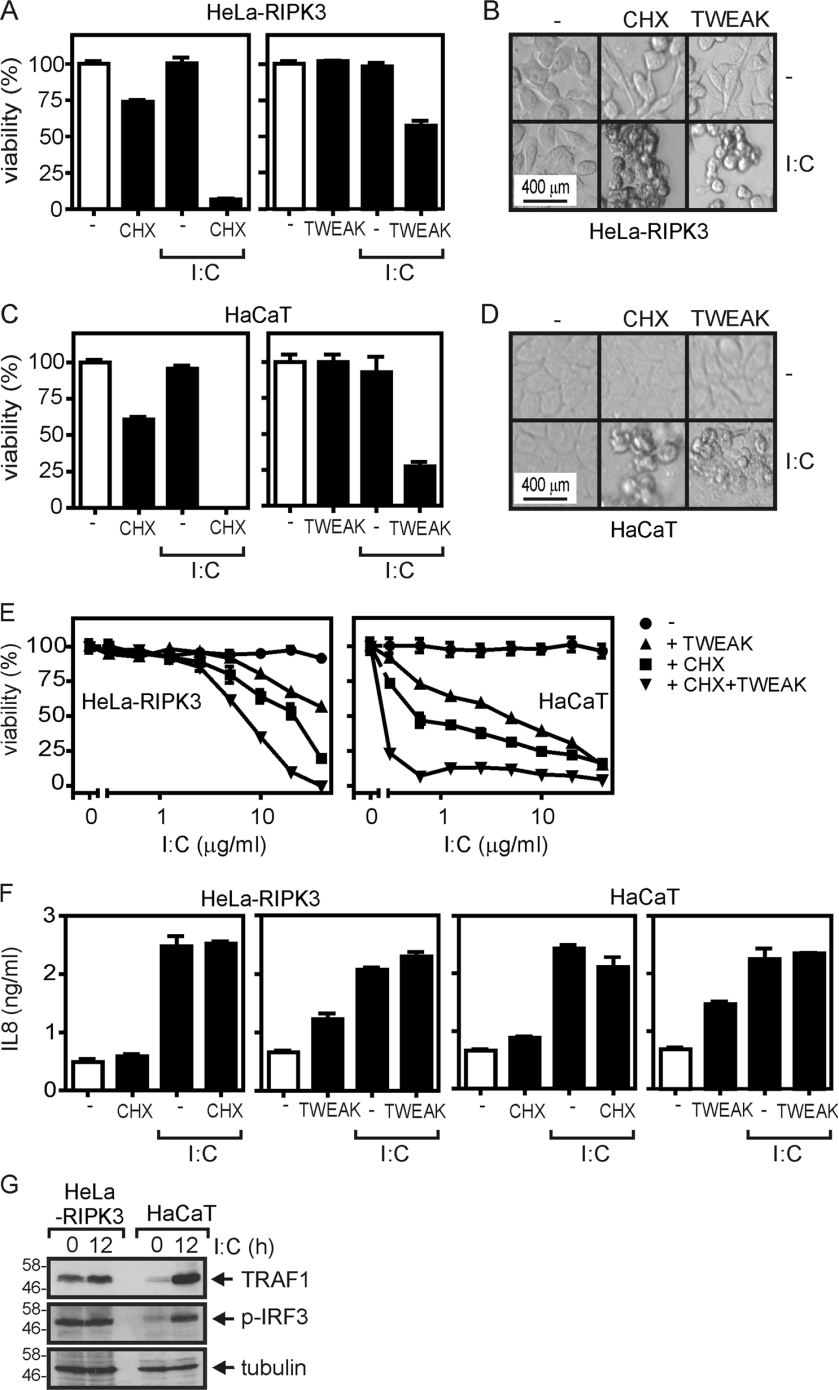
Cycloheximide and TWEAK sensitize for poly(I:C)-induced cell death. **a**–**d** Hela-RIPK3 and HaCaT cells were stimulated with the indicated combinations of poly(I:C) (40 µM), Flag-TWEAK (200 ng/ml), and CHX (2.5 µg/ml). Next day, cellular viability was quantified by crystal violet staining (**a**, **c**) and documented by microscopy (**b**, **d**). **e** Cells were challenged with the indicated concentrations of poly(I:C) in the presence and absence of 200 ng/ml of Flag-TWEAK, 2.5 µg/ml CHX, or a combination of both. One day later, cellular viability was again quantified by help of crystal violet staining. **f** Cells were challenged in triplicates with poly(I:C) in the presence of the indicated combinations of CHX (2.5 µg/ml) and Flag-TWEAK (200 ng/ml). Next day, supernatants were analyzed with respect to their IL8 content by ELISA. **g** Cells were stimulated as indicated for 12 h with poly(I:C) and total cell lysates were analyzed for the expression of the indicated proteins by western blotting

In HeLa-RIPK3 transfectants and HaCaT cells, poly(I:C) alone induced no or only moderate cell death (Fig. 1a–d). In the presence of soluble Flag-tagged TWEAK (Flag-TWEAK, ref. ^7^), however, there was regularly enhanced cell death induction (Fig. 1a–d). It is very well established that treatment with cycloheximide (CHX) sensitizes many cell types, including HeLa and HaCaT cells, for death receptor-induced cell death. Indeed, CHX treatment also sensitized HeLa-RIPK3 and HaCaT cells for poly(I:C)-induced cell death (Fig. 1a–d), and this cytotoxic response was further enhanced by stimulation with Flag-TWEAK (Fig. 1e). Noteworthy, poly(I:C) efficiently triggered proinflammatory signaling independently from treatment with CHX or Flag-TWEAK what is evident from the upregulation of the chemokine interleukin (IL)-8 and the nuclear factor-κB (NF-κB)-regulated survival protein TRAF1 (Fig. 1f, g). Thus, the need of TWEAK or CHX treatment to uncover robust poly(I:C)-induced cell death is response-specific and does not reflect a general TWEAK/CHX-dependency of poly(I:C)-induced signaling in HeLa- and HaCaT cells.

**Fig. 2:**
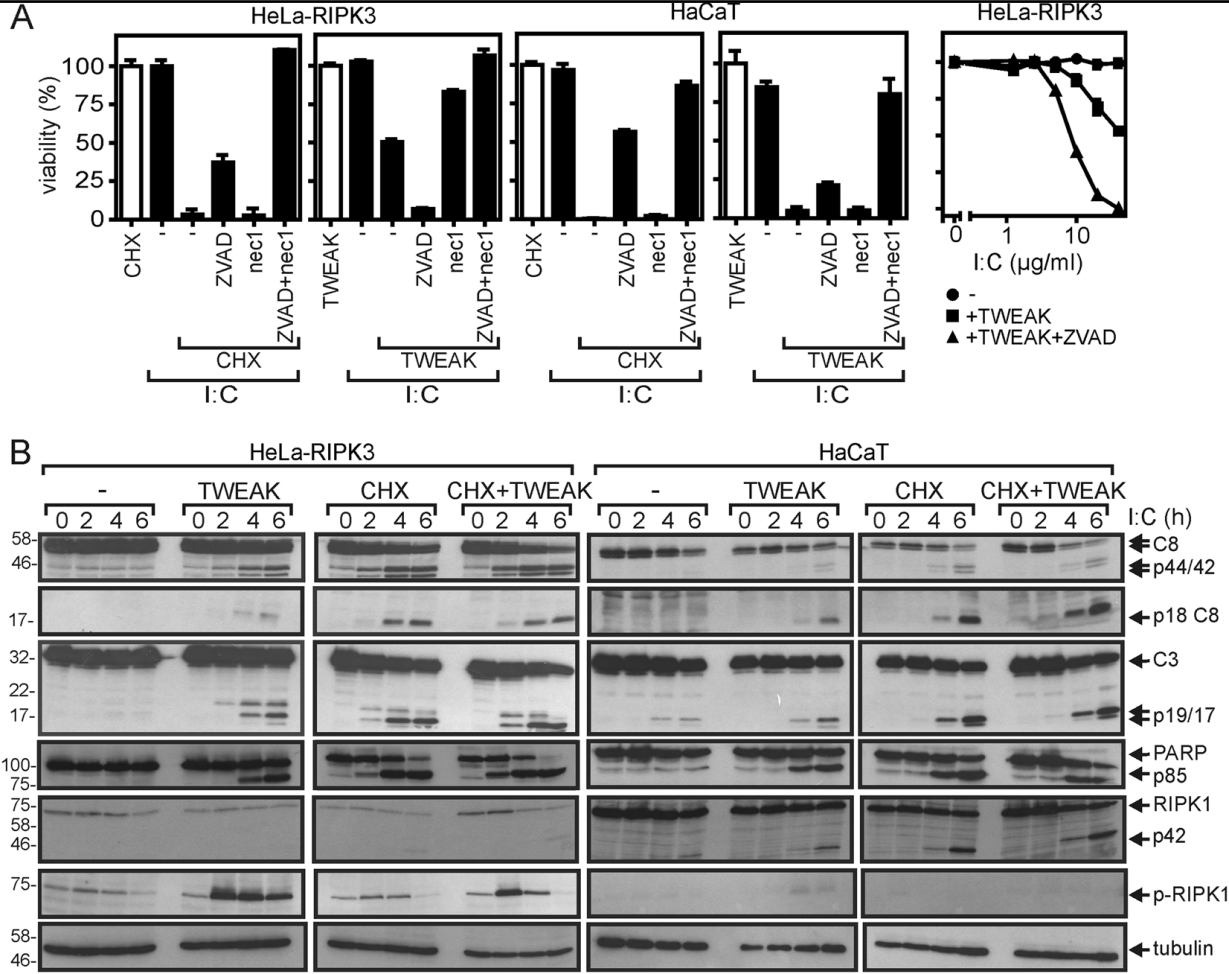
Poly(I:C) triggers apoptotic and necroptotic cell death. **a** HeLa-RIPK3 and HaCaT cells were challenged overnight as indicated with poly(I:C) (40 µg/ml), CHX (2.5 µg/ml), Flag-TWEAK (200 ng/ml), ZVAD (20 µM), and necrostatin-1 (90 µM). Finally, cellular viability was evaluated by help of crystal violet staining. To allow a better evaluation of the sensitizing effect of ZVAD on poly(I:C)/TWEAK-induced cell death in HeLa-RIPK3 cells, a dose–response experiment with decreasing concentrations of poly(I:C) was performed (right panel). **b** Cells were challenged with 40 µg/ml poly(I:C) in the presence and absence of CHX (2.5 µg/ml) or Flag-TWEAK (200 ng/ml) and finally total cell lysates were subjected to western blotting to detect the indicated proteins

Death receptors can induce cell death by two biochemically distinct programs, caspase-8-mediated apoptosis, and RIPK1-, RIPK3-, and MLKL-dependent necroptosis. Notably, although TWEAK enhances both TNF-induced necroptosis and apoptosis^7,9,19–22^, it preferentially enhances TRAIL death receptor-induced necroptosis^21^. We therefore investigated in more detail whether TWEAK affects poly(I:C)-induced apoptosis and/or necroptosis. A varying contribution of apoptosis and necroptosis to poly(I:C)-induced killing in CHX- and Flag-TWEAK-sensitized cells was evident from inhibitor studies with zVAD-fmk (ZVAD) and necrostatin-1 (nec1). Although the pan-caspase inhibitor ZVAD rescues cells from death receptor-induced apoptosis, nec1 inhibits the kinase activity of RIPK1, which has a crucial role in triggering necroptosis by various inducers^23^. Treatment with ZVAD alone significantly inhibited poly(I:C)-induced cell death both in CHX-sensitized HeLa-RIPK3 and HaCaT cells (Fig. 2a, first and third panel). ZVAD, however, enhanced poly(I:C)-induced killing in Flag-TWEAK-treated HeLa-RIPK3 cells but had again a partial inhibitory effect on cell death induction in FLAG-TWEAK-treated HaCaT cells (Fig. 2a, second and fourth panel). Nec1 alone showed a protective effect on Flag-TWEAK-sensitized HeLa-RIPK3 cells but otherwise failed to elicit an effect on the cytotoxic response (Fig. 2a, second panel). A combination of ZVAD and nec1, however, was fully protective, against poly(I:C)-induced cell death irrespective of the cell type and the sensitizer considered (Fig. 2a). Thus, the ZVAD/nec1 mixture was superior to ZVAD with respect to the rescue from poly(I:C)-induced cell death. Treatment with CHX enhanced poly(I:C)-induced processing of caspases better than Flag-TWEAK, whereas the latter enabled poly(I:C) to trigger RIPK1 phosphorylation at serine 166 much more efficiently than CHX (Fig. 2b). Intriguingly, although cotreatment with CHX and Flag-TWEAK was neutral to superior to the single treatments with respect to poly(I:C)-induced caspase processing, the sensitizing effect of Flag-TWEAK on poly(I:C)-induced RIPK1 phosphorylation was antagonized by CHX (Fig. 2b). The latter observation corresponds very well to the fact that apoptotic caspases inhibit necroptosis, e.g., by cleavage of RIPK1 and RIPK3^24^.

### Soluble TWEAK sensitizes for poly(I:C)-induced cell death independent from TNF

**Fig. 3:**
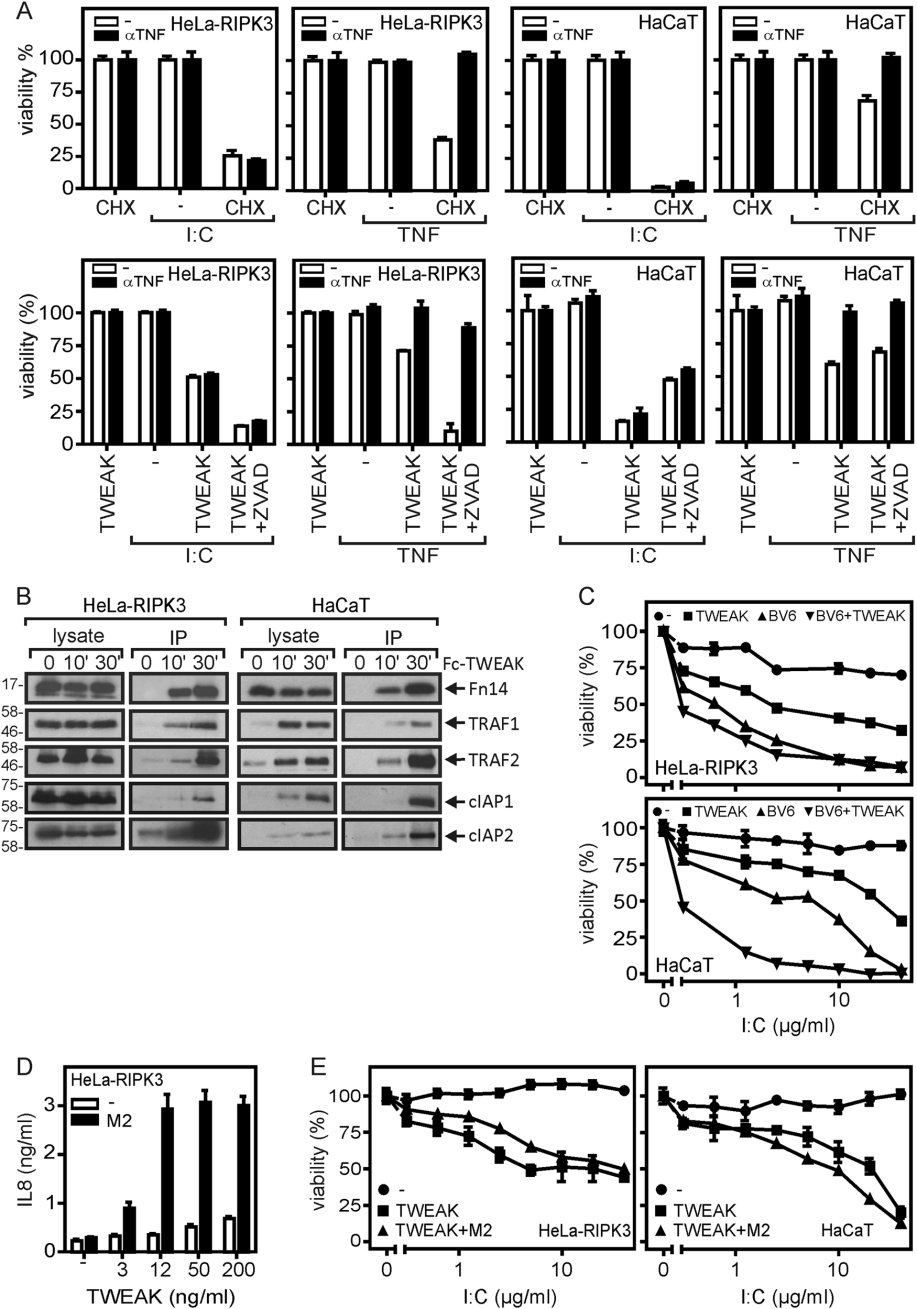
Poly(I:C)-induced cell death is independent from TNF. **a** HeLa-RIPK3 and HaCaT cells were treated overnight with the indicated mixtures of poly(I:C) (40 µg/ml), CHX (2.5 µg/ml), Flag-TWEAK (200 ng/ml), ZVAD (20 µM), TNF (100 ng/ml), and anti-TNF and control IgG1 (both 25 µg/ml). Finally, cellular viability was determined by crystal violet staining. **b** Cells were stimulated with Fc-Flag-TWEAK (2 µg/ml), a fusion protein of soluble TWEAK with an N-terminal Fc domain. TWEAK-bound Fn14 and Fn14-associated signaling proteins were immunoprecipitated with protein G beads. **c** Hela-RIPK3 and HaCaT cells were stimulated with the indicated concentrations of poly(I:C) in the presence and absence of BV6 (10 µM) and/or Flag-TWEAK (200 ng/ml). Cell viability was finally measured again using crystal violet staining. **d** HeLa-RIPK3 cells were challenged with the indicated concentrations of Flag-TWEAK in the presence and absence of the anti-Flag mAb M2 (1 µg/ml). Next day, IL8 production was evaluated by ELISA assay. **e** HeLa-RIPK3 cells were stimulated with poly(I:C) and Flag-TWEAK (200 ng/ml) in the presence and absence of M2 (1 µg/ml), and were analyzed the next day for cellular viability

In a small subset of tumor cell lines, TWEAK induces apoptosis by triggering TNF production and subsequent stimulation of TNFR1^7,8^. To evaluate the possible relevance of this mechanism for poly(I:C)/TWEAK-induced cell death, we tested the effect of a TNF-blocking antibody. The latter efficiently inhibited TNF-induced cell death but showed no effect on poly(I:C)/TWEAK-induced cell death (Fig. 3a). This suggests that the enhancing effect of Flag-TWEAK on poly(I:C)-induced cell death is primarily related to the ability of TWEAK to deplete the cytosolic available pool of protective TRAF2-cIAP1/2 complexes^8,9^. Not unexpected, the sensitizing effect of CHX on poly(I:C)-induced cell death was TNF-independent as well (Fig. 3a). In accordance with this idea, Flag-TWEAK stimulation of HeLa-RIPK3 and HaCaT cells resulted in efficient recruitment of TRAF2, cIAP1, and cIAP2 to Fn14 (Fig. 3b). Moreover, there was also efficient recruitment of TRAF1 that forms heterotrimers with TRAF2, which recruit cIAP1 even better then TRAF2 homotrimers^25^. The cIAP1 and cIAP2 degradation-inducing IAP antagonist BV6^26^ sensitized HeLa-RIPK3 and HaCaT cells in a similar and even more effective manner than Flag-TWEAK for poly(I:C)-induced cell death (Fig. 3c).

Similar to other ligands of the TNFSF, TWEAK occurs as a soluble and as membrane-bound molecule (memTWEAK). Both TWEAK variants interact with Fn14 but noteworthily trigger different states of Fn14 activity. Although soluble TWEAK and memTWEAK similarly engage activation of the alternative NF-κB pathway, membrane TWEAK is superior to soluble TWEAK in the activation of the classical NF-κB pathway resulting in the production of proinflammatory cytokines such as IL8^27^. To clarify whether the type of TWEAK species is of relevance for Fn14-mediated sensitization for poly(I:C)-induced cell death, we compared the effect of Flag-TWEAK in the presence and absence of the anti-Flag mAb M2. It has been shown that M2-oligomerized soluble Flag-TWEAK mimic the activity of memTWEAK^27,28^. Accordingly, there was only a very weak increase in IL8 production in HeLa-RIPK3 cells treated with Flag-TWEAK, whereas IL8 production was strongly upregulated in response to M2-oligomerized Flag-TWEAK (Fig. 3d). Anti-Flag oligomerization, however, had no effect on the cell death enhancing effect of Flag-TWEAK (Fig. 3e). Thus, already in its soluble form, TWEAK exerts its maximal enhancing activity on poly(I:C)-induced cell.

### Apoptosis and necroptosis induction by poly(I:C) require RIPK1 while only apoptosis induction depend also on FADD and caspase-8

**Fig. 4:**
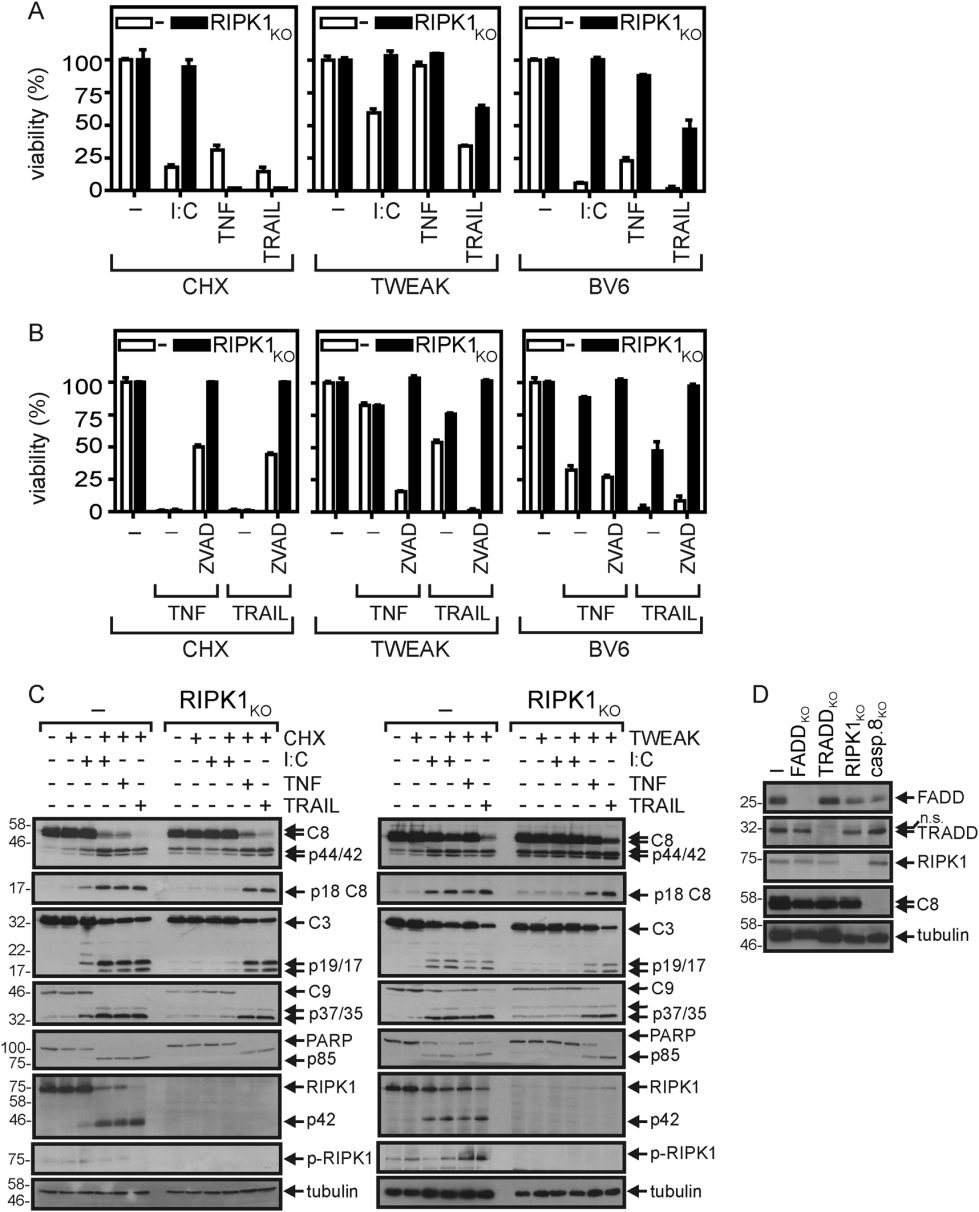
RIPK1 is essential for poly(I:C)-induced cell death. **a**, **b** HeLa-RIPK3 and HeLa-RIPK3-RIPK1_KO_ cells were stimulated as indicated with poly(I:C) (40 µg/ml), Flag-TWEAK (200 ng/ml), CHX (2.5 mg/ml), ZVAD (20 µM), TNF (100 ng/ml), TRAIL (100 ng/ml), and necrostatin-1 (90 µM). Next day, cellular viability was determined by crystal violet staining. **c** HeLa-RIPK3 (−) and HeLa-RIPK3-RIPK1_KO_ cells were stimulated with poly(I:C) (40 µg/ml), TNF (100 ng/ml), and TRAIL (100 ng/ml) in the presence of Flag-TWEAK (200 ng/ml) or CHX (2.5 µg/ml). After 6 h, total cell lysates were prepared and analyzed by western blotting. **d** HeLa-RIPK3 (−) cells and variants derived thereof lacking expression of FADD, TRADD, RIPK1, and caspase-8 were analyzed by western blotting with respected to the indicated proteins

To verify the relevance of the cytosolic death domain-containing proteins TRADD, FADD, and RIPK1, and of caspase-8 in poly(I:C)/CHX and poly(I:C)/TWEAK-induced cell death in more detail, we investigated a panel of HeLa-RIPK3 cells lacking expression of each of these molecules. Cell death induction by poly(I:C) was completely abrogated in the absence of RIPK1 irrespective of whether CHX, TWEAK, or BV6 have been used to enhance the cell death response (Fig. 4a). In contrast, TRAIL death receptor-induced cell death was still evident in the HeLa-RIPK3-RIPK1_KO_ cells under all three conditions (Fig. 4a). TNF/CHX-induced cell death was even enhanced in the RIPK1-deficient HeLa-RIPK3 cells (Fig. 4a). In accordance with the established fact that RIPK1 is obligate for TNF- and TRAIL-induced necroptosis, HeLa-RIPK3-RIPK1_KO_ cells stimulated with TRAIL or TNF and sensitized for cell death induction by CHX, TWEAK, or BV6 could only die by apoptosis, as cell death was completely prevented by caspase inhibition (Fig. 4b). RIPK1 was required for apoptosis induction by poly(I:C) as the absence of RIPK1 prevented caspase activation, irrespective of whether CHX or ZVAD has been used for sensitization (Fig. 4c, d). In contrast, RIPK1 was dispensable for caspase activation/apoptosis induction by TRAIL and TNF (Fig. 4c, d).

**Fig. 5:**
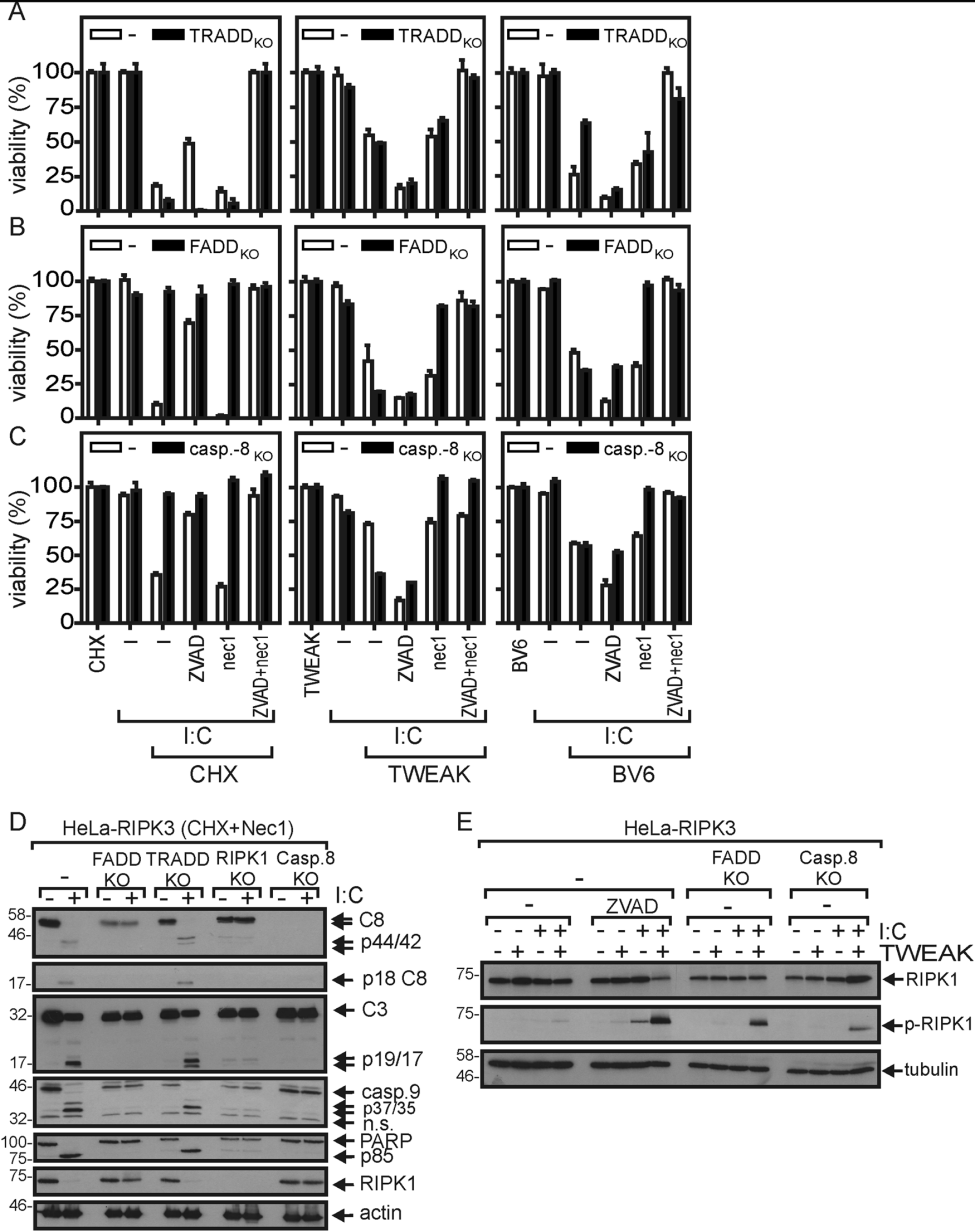
FADD and caspase-8 are required for poly(I:C)-induced apoptosis. **a-c** HeLa-RIPK3 (−) (**a**–**c**), HeLa-RIPK3-TRADD_KO_ (**a**), HeLa-RIPK3-FADD_KO_ (**b**), and HeLa-RIPK3-Casp8_KO_ (**c**) cells were challenged as indicated with poly(I:C) (40 µg/ml), CHX (2.5 µg/ml), Flag-TWEAK (200 ng/ml), BV6 (10 µM), neccrostatin-1 (90 µM), and 20 µM ZVAD. Next day, cellular viability was evaluated. **d** Cells were stimulated for 6 h with poly(I:C) (40 µg/ml) in the presence of CHX (2.5 µg/ml) and neccrostatin-1 (90 µM). Total cell lysates were analyzed by western blotting. **e** Cells were treated for 6 h with poly(I:C) (40 µg/ml) in the presence and absence of Flag-TWEAK (200 ng/ml) and, where indicated, with ZVAD (20 µM). Total cell lysates were analyzed by western blotting

TRADD deficiency sensitized CHX-treated HeLa-RIPK3 cells for poly(I:C)-induced necroptosis but showed no major effect on poly(I:C)/TWEAK-induced cell death (Fig. 5a). FADD and caspase-8 deficiency abrogated caspase activation and apoptosis induction by poly(I:C)/CHX (Fig. 5b–d) but showed no or even a mild sensitizing effect on poly(I:C)/TWEAK- and poly(I:C)/BV6-induced necroptosis (Fig. 5b, c). Noteworthy, in contrast to RIPK1 deficiency, treatment with the RIPK1 kinase inhibitor nec1 failed to inhibit poly(I:C)-induced activation of caspases in CHX-treated cells (Fig. 5d). This suggests that RIPK1 can act independent from its kinase activity as a scaffold protein in poly(I:C)-induced caspase-8 activation/apoptosis. Phosphorylation of RIPK1 at serine 166 was evident in poly(I:C)/TWEAK-treated HeLa-RIPK3-FADD_KO_ and HeLa-RIPK3-casp8_KO_ cells, as it was in HeLa-RIPK3 cells when pretreated with ZVAD (Fig. 5e). This is in good accordance with the established fact that caspases inhibit necroptosis^29^.

### TRADD and RIPK1 are required for poly(I:C)-induced TRAF1 expression but are dispensable for upregulation of IL8

**Fig. 6:**
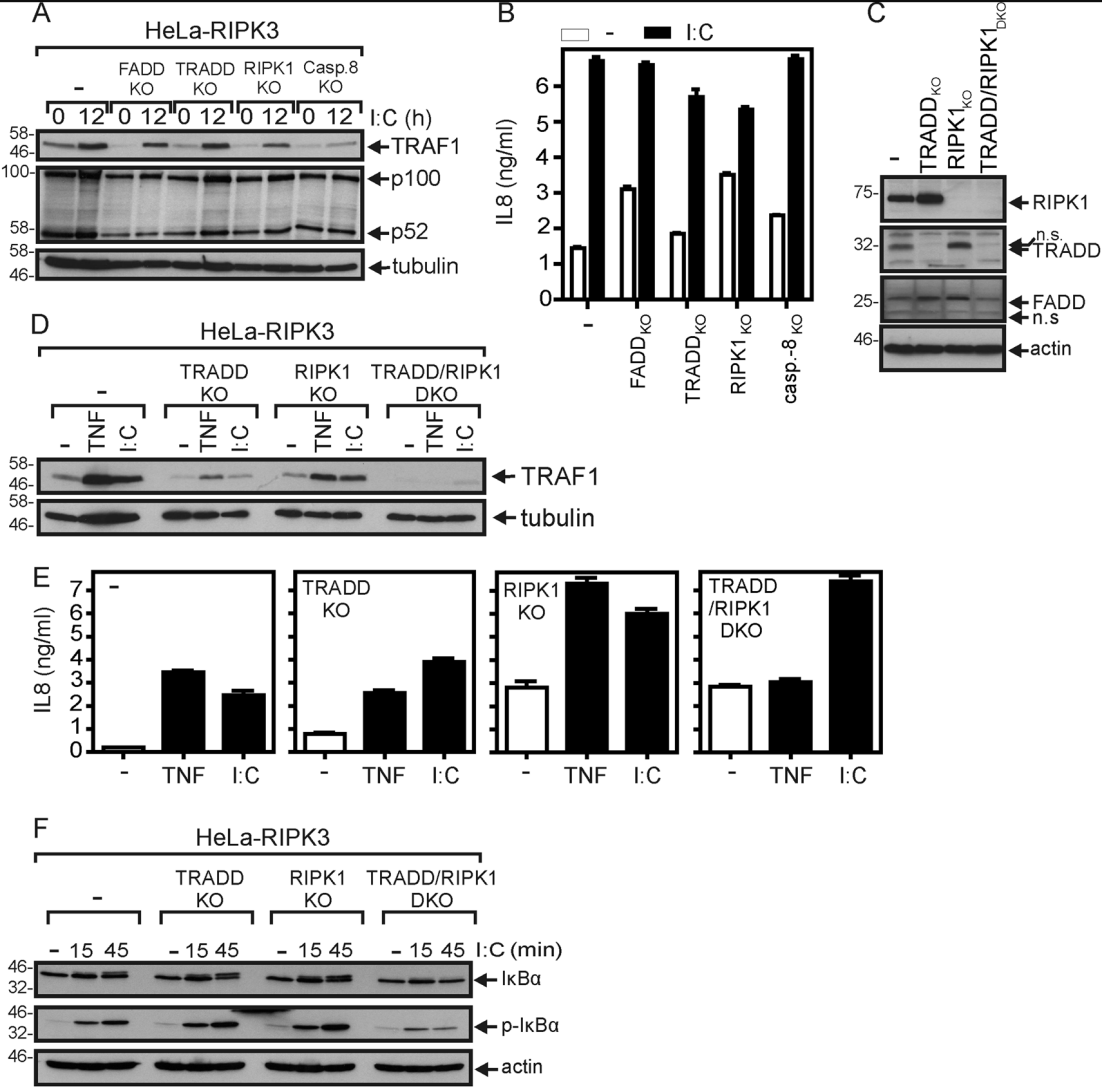
TRADD/RIPK1 double-deficiency attenuates proinflammatory poly(I:C) signaling. **a**, **b** The various HeLa-RIPK3 variants were stimulated for 12 h (**a**) or overnight (**b**) with 40 µM poly(I:C). Total cell lysates were analyzed by western blotting (**a**) and cell culture supernatants were analyzed by ELISA for their IL8 content (**b**). **c** HeLa-RIPK3 (−), HeLa-RIPK3-TRADD_KO_, HeLa-RIPK3-RIPK1_KO_, and HeLa-RIPK3-TRADD/RIPK1_DKO_ cells were analyzed by western blotting for the expression of the indicated proteins. **d**, **e** The various cell variants were stimulated with 40 µM poly(I:C) or 100 ng/ml TNF and were then investigated as in “**a**” in “**b**” for induction of TRAF1 (**d**) and IL8 (**e**). **f** Cells were treated with 10 µM of the NEDD8-activating enzyme inhibitor MLN4924 to prevent degradation of phosphorylated IκBα and were stimulated for the indicated times with 40 µM poly(I:C). Total cell lysates were analyzed for the presence of the indicated protein species

Both poly(I:C)-induced upregulation of the cytokine IL8, which is controlled by NF-κB signaling and various MAP kinase cascades, and of TRAF1, a well-established target of the classical and alternative NF-κB pathway, remained principally intact in all HeLa-RIPK3 variants (Fig. 6a, b). TRAF1 induction by poly(I:C), however, was regularly reduced in HeLa-RIPK3-casp8_KO_ cells (Fig. 6a). Neither poly(I:C) treatment per se nor deficiency of TRADD, FADD, RIPK1, or caspase-8 showed an effect on p100 processing, suggesting that the alternative NF-κB signaling pathway has no major role in poly(I:C) signaling in HeLa-RIPK3 cells (Fig. 6a). As we noticed in other studies (Füllsack et al., submitted) that TRADD and RIPK1 redundantly act in TNF-induced proinflammatory signaling, we also analyzed TRADD/RIPK1-double-deficient HeLa-RIPK3 cells for gene induction by poly(I:C) (Fig. 6c). As expected, TNF-induced expression of TRAF1 and IL8 was blunted in HeLa-RIPK3-TRADD/RIP_DKO_ cells but not in the single deficient variants (Fig. 6d, e). With respect to gene induction by poly(I:C) the situation was more complex. There was at best residual poly(I:C)-induced TRAF1 expression in HeLa-RIPK3-TRADD/RIPK1_DKO_ cells (Fig. 6d) and IκBα phosphorylation, an early event in classical NF-κB signaling, was strongly reduced too (Fig. 6f). In variance to TNF, however, poly(I:C) still upregulated IL8 production in the double-deficient HeLa-RIPK3 variant (Fig. 6e). The finding that poly(I:C) in contrast to TNF upregulates IL8 production in HeLa-RIPK3-TRADD/RIPK1_DKO_ cells may simply reflect the fact that IL8 production can also be stimulated via the IRF-3/ISRE pathway^30,31^ but this was not further investigated in the current study.

### FLIP proteins protect from poly(I:C)-induced cell death

**Fig. 7:**
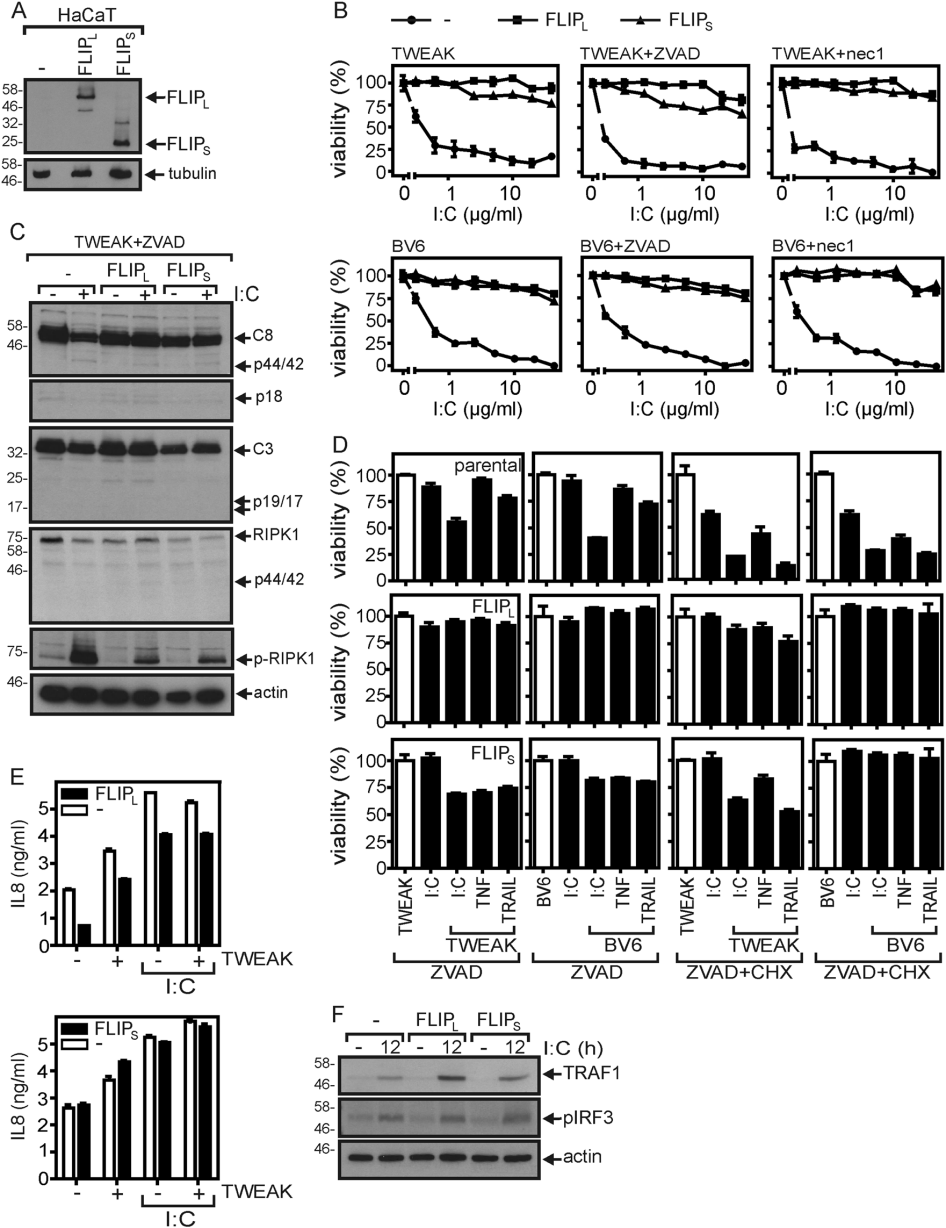
FLIP proteins inhibit poly(I:C)-induced cell death. **a** HaCaT (−), HaCaT-FLIP_L_, and HaCaT-FLIP_S_ were analyzed by western blotting for FLIP_L_ and FLP_S_ expression. **b** The various HaCaT variants were stimulated with increasing concentrations of poly(I:C) in the presence of the indicated combinations of Flag-TWEAK (200 ng/ml), BV6 (10 µM), nec1 (90 µM), and ZVAD (20 µM). Next day, cellular viability was determined by crystal violet staining. **c** Cells were stimulated for 6 h as indicated with of poly(I:C) (40 µg/ml) in the presence of Flag-TWEAK (200 ng/ml) and ZVAD (20 µM). Total cell lysates were analyzed for the presence of the indicated proteins by western blotting. **d** Cells were stimulated with the indicated mixtures of poly(I:C), TNF, TRAIL, Flag-TWEAK, and BV6 in the presence of ZVAD and CHX. Next day, cells were again analyzed for their viability. **e**, **f** The various HaCaT variants were stimulated for 12 h (**f**) or overnight (**e**) with 40 µg/ml poly(I:C) and were analyzed by western blotting (**f**) and IL8 ELISA (**e**). Where indicated in “**e**”, cells were treated in the presence of 200 ng/ml Flag-TWEAK

FLIP_S_, the short isoform of the enzymatically compromised caspase-8 homolog FLIP, acts as an inhibitor of death receptor-induced apoptosis^29^ but can also promote necroptosis^32,33^. The long isoform of FLIP (FLIP_L_) furthermore acts expression level-dependent as a promoter or inhibitor of death receptor-induced apoptosis^29^. Analysis of HaCaT cells stably transfected with FLIP_S_ and FLIP_L_, respectively, revealed that both FLIP isoforms inhibited poly(I:C)/TWEAK-induced cell death (Fig. 7a, b). In line with our previous observation that TWEAK mainly enhances necroptotic poly(I:C) signaling, poly(I:C)/TWEAK-induced phosphorylation of RIPK1 was significantly reduced in the FLIP_L/S_ transfectants as well (Fig. 7c). A similar anti-necroptotic activity of the two FLIP isoforms was also evident when TWEAK- or BV6-sensitized cells were challenged in the presence of ZVAD (or ZVAD + CHX) with TRAIL or TNF (Fig. 7d). Gene induction by poly(I:C), however, remained largely intact in the FLIP_L/S_ expressing HaCaT variants (Fig. 7e, f). We also established HeLa-RIPK3 variants with stable expression of FLIP_S_ and FLIP_L_ (Supplementary Data Fig. S1a). Although not as efficient as the HaCaT variants, both HeLa-RIPK3-FLIP_S_ and HeLa-RIPK3-FLIP_L_ cells were found to be protected from poly(I:C)-triggered cell death irrespective of the sensitizer (BV6 or TWEAK) used (Supplementary Data Fig. S1b). Moreover, FLIP_S_ and FLIP_L_ expression improved survival of poly(I:C)/TWEAK- and poly(I:C)/BV6-treated cells in the presence of ZVAD, as well as in the presence of nec1 (Supplementary Data Fig. S1b). Similar to the HaCaT-FLIP_S/L_ transfectants, the HeLa-RIPK3-FLIP_S/L_ transfectants also showed reduced RIPK1 phosphorylation in response to poly(I:C))/TWEAK in ZVAD-treated cells (Supplementary Data Fig. S1c). Poly(I:C)-induced IL8 production was not affected in HeLa-RIPK3-FLIP_L_ cells and was also intact in HeLa-RIPK3-FLIP_S_ cells (Supplementary Data Fig. S1d). In the latter case, basal and poly(I:C)-induced IL8 production was higher than in the HeLa-RIPK3 control cells. However, whether this reflects the known NF-κB-stimulating activity of FLIP proteins or a specific effect of FLIP_S_ on poly(I:C) signaling was not further investigated. In sum, FLIP_S_ and FLIP_L_ showed similar effects on poly(I:C) signaling both in HeLa-RIPK3 and HaCaT cells.

## Discussion

Fn14 is highly and dynamically expressed during embryonal development, but in the healthy adult organism Fn14 expression is low and restricted to very few cell types, e.g., mesenchymal progenitor cells and cells of the kidney^1^. Fn14 expression, however, is strongly induced in a variety of non-hematopoietic cell types after tissue injury^1^. The latter is intimately linked with the release of PAMPs/DAMPs such as lipopolysaccharide (LPS), double-stranded RNA, which is generated during viral replication, and intracellular molecules released from destroyed cells (e.g., ATP)^34^. Innate immunity cells, but also many non-immune cell types, sense PAMPs and DAMPs by help of membranous and cytosolic receptors pattern recognition receptors (PRRs)^34^. Double-stranded RNA for example is recognized by the transmembrane receptor TLR3 but also by cytosolic RIG1-like receptors^35,36^. PRRs stimulate proinflammatory signaling pathways but may also trigger apoptotic and necroptotic cell death under certain circumstances^34–36^. Thus, PRRs and the TWEAK/Fn14 system gain relevance under similar conditions and control an overlapping set of cellular activities.

In recent years, we showed that death receptor-induced cell death can be strongly enhanced by TWEAK^7,9,19–21^. FADD, TRADD, RIPK1, and caspase-8 transduce apoptotic and necroptotic death receptor signaling but are also of relevance for proinflammatory signaling by these receptors. As these molecules have also been implicated in poly(I:C)-induced signaling^32,37–45^, we wondered whether TWEAK is also able to modulate poly(I:C)-induced cell death. We investigated this in HaCaT and HeLa cells whose poly(I:C) responsiveness is well documented^32,43–46^. Accordingly, poly(I:C) induced production of the proinflammatory cytokine IL8 both in HaCaT cells and HeLa transfectants expressing RIPK3 (Fig. 1). Treatment with poly(I:C) alone neither induced cell death in HaCaT cells nor in HeLa-RIPK3 cells, unless cells were sensitized with CHX or TWEAK (Fig. 1). Notably, poly(I:C)/CHX mainly induced caspase-dependent apoptotic cell death, while poly(I:C)/TWEAK preferentially triggered caspase-independent necroptotic cell death (Fig. 2). This suggests that the two sensitizers CHX and TWEAK target different mechanisms protecting cells from poly(I:C)-induced killing. Indeed, the cell death-sensitizing effect of CHX in context of death receptor signaling has been mainly traced back to reduced expression of the caspase-8 inhibitory FLIP proteins^47,48^, whereas TWEAK depletes the cytosolic pool of TRAF2-cIAP1 and TRAF2-cIAP2 complexes that dampen death receptor-induced necroptotic activity of RIPK1 by cIAP1/2-mediated K63 ubiquitination^14^. In accordance with a similar mode of action of CHX and TWEAK in poly(I:C)-induced signaling, we observed strongly reduced poly(I:C)-induced cell death in FLIP_L_ and FLIP_S_ transfectants (Fig. 7b–d, S1b), and enhanced cell death induction in the presence of the cIAP antagonist BV6 (Fig. 3c)^26^.

Analysis of HeLa-RIPK3 transfectants lacking RIPK1, FADD, TRADD, or caspase-8 expression demonstrated a crucial role of FADD, caspase-8, and RIPK1 in poly(I:C)-induced cell death. Although FADD and caspase-8 turned out to be essential for poly(I:C)-induced caspase activation (Fig. 5d) and apoptosis (Fig. 5b, c), these molecules were dispensable for the necroptotic poly(I:C) response (Fig. 5b–e). FADD, caspase-8, and RIPK1 have also a crucial role in TNF- and TRAIL-induced cell death but there are clear differences in the way how poly(I:C), TNF, and TRAIL utilizes these proteins for death induction even in the same cellular system (HeLa-RIPK3 cells). First, although RIPK1 is essential for both apoptosis and necroptosis induction by poly(I:C), in TNF and TRAIL signaling, it is only obligate for necroptosis (Fig. 4a). Second, FADD and caspase-8 are of crucial relevance for both TRAIL-induced apoptosis and TRAIL-induced necroptosis (Füllsack et al., submitted), in context of poly(I:C)- and TNF-induced cell death; however, these two molecules are dispensable for necroptosis (Fig. 5b, c and Füllsack et al., submitted). Evaluation of HeLa-RIPK3 cells lacking TRADD and RIPK1 expression revealed furthermore that these two proteins are dispensable for poly(I:C)-induced IL8 expression despite having a redundant but obligate role in the corresponding response to TNF and TRAIL (Fig. 6, Füllsack et al., submitted). The different roles of TRADD, RIPK1, FADD, and caspase-8 in poly(I:C), TNF, and TRAIL-induced signaling could be largely explained by considering two issues: first, that RIPK1 and TRADD on the one side and FADD and caspase-8 on the other side act in reverse order in TNF- (or poly(I:C)) and TRAIL-induced signaling; second, that the TRADD/RIPK1 dyad triggers necroptosis and proinflammatory signaling, while the FADD/caspase-8 dyad stimulates apoptosis; and third, that the TRADD/RIPK1 and FADD/caspase-8 dyad mutually activate each other if one of both has been engaged by appropriate receptors, e.g., TLR3, TNFR1, and TRAIL death receptors. The RIPK1/TRADD dyad act in this model upstream of the FADD/caspase-8 dyad in TNF and poly(I:C) signaling but downstream of the latter in TRAIL signaling. The FADD/caspase-8 dyad is therefore dispensable for RIPK1-mediated necroptosis and proinflammatory signaling by polyI:C and TNF but required in the case of TRAIL as an adapter to link the TRAIL death receptors with the TRADD/RIPK1 dyad. Vice versa, the downstream location of the FADD/caspase-8 dyad in poly(I:C) and TNF signaling explains the requirement of RIPK1 and/or TRADD for caspase-8 activation and apoptosis. The fact that RIPK1 deficiency is already sufficient to shut down the apoptotic poly(I:C) response while blockade of TNF-induced apoptosis signaling requires RIPK1/TRADD double deficiency (Füllsack et al., submitted) might simply reflect that TNFR1 strongly interact with both molecules, whereas the poly(I:C) receptor TLR3, in a TRIF-dependent manner, preferentially recruits RIPK1 but TRADD only indirectly via RIPK1^40^. Interestingly, deficiency of RIPK1 but not inhibition of its kinase activity abrogated poly(I:C)-induced activation of caspases in CHX-treated cells (Fig. 5d). Thus, RIPK1 seems to act independent from its kinase activity as a scaffold protein in poly(I:C)-induced caspase-8 activation/apoptosis. This mode of apoptosis induction is not without precedence and has also been described in context of endoplasmic reticulum (ER) stress-induced cell death and in models of RIPK3-mediated apoptosis^44,49,50^. In context of TNFR1, however, caspase-8 activation/apoptosis occurs either RIPK1-independent or RIPK1-dependent under involvement of its kinase activity^51^. It will be interesting to see in the future whether the differential relevance of RIPK1 kinase activity in poly(I:C)- and TNF-induced RIPK1-mediated apoptosis reflects a special role of TRADD in apoptotic TNF signaling.

We found that FLIP_L_ and FLIP_S_ both prevent apoptotic and necroptotic poly(I:C)-induced cell death in HaCaT cells (Fig. 7), and attenuated these responses in HeLa-RIPK3 cells (Supplementary Fig. S1). The anti-apoptotic effect of these molecules is certainly not surprising in view of their well-established inhibitory effect on FADD-mediated caspase-8 maturation. As caspase-8 furthermore inhibits necroptosis by cleavage of RIPK1 and RIPK3, the anti-necroptotic activity of the FLIP proteins imply that these molecules have also a yet poorly defined caspase activity-independent survival function. Another study reported a differential effect of the two FLIP isoforms on necroptosis induction by poly(I:C), whereby FLIP_S_ expression resulted in enhanced necroptosis^32^. Notably, FLIP_L_-caspase-8 heterodimers, in contrast to FLIP_S_-caspase-8 heterodimers, allows the first of the two caspase-8 maturation steps resulting in a complex with a limited substrate spectrum, which still covers RIPK1 and RIPK3. Thus, the RIPK1/RIPK3 inhibitory potential of FLIP_L_ seems to be higher than that of FLIP_S_. It appears therefore possible that the reported necroptosis “sensitizing” effect of ectopic FLIP_S_ expression does not reflect an active necroptosis promoting role of this isoform but rather the competitive inhibition of the superior endogenously expressed necroptosis inhibitor FLIP_L_. This implies that besides caspase-8-dependent cleavage of RIPK1 and RIPK3, which is only promoted by FLIP_L_, there is another FLIP_L_ and FLIP_S_ common mechanism by which these proteins interfere with necroptosis. Indeed, both FLIP variants interact with the NEMO/IKKγ subunit of the IKK complex, which regulates NF-κB signaling and RIPK1 activity^52^. Thus, at the first glance, contradictory effects of FLIP_S_ on poly(I:C)-induced necroptosis in our study and Feoktistova et al.^32^ may reflect the complex connectivity of caspase-8, FLIP_L/S_, RIPK1, and other IKK-related activities in context of cell death signaling, which may allow subtle changes in expression levels to result in different net effects.

## Material and methods

### Cell lines, reagents, and antibodies

HeLa-RIPK3 cells were a kind gift from Professor Martin Leverkus (University of Aachen, Germany) and are described in Karl et al.^21^. The generation and basic characterization of the various HeLa-RIPK3 knockout variants has been described elsewhere (Füllsack et al., submitted). HeLa-RIPK3 and HeLa-RIPK3 knockout variants were sustained in RPMI1640 medium containing 10% heat-inactivated fetal bovine serum (FBS). HeLa-RIPK3-FLIP_L/S_ transfectants were generated by sleeping beauty transposon-based expression constructs encoding FLIP_L_ and FLIP_S_. HaCaT cells were maintained in Dulbecco’s modified Eagle’s medium supplemented with 10% FBS. HaCaT cells stably transfected with FLIP_L_ or FLIP_S_ have been described in Kavuri et al.^52^. All cells were grown at 37 °C and 5% CO_2_.

Poly(I:C) and CHX were provided by Sigma-Aldrich (Deisenhofen, Germany). The SMAC mimetic BV6 was obtained by Syngene (Bangalore, India), zVAD-fmk was from Thermo Fisher Scientific (Waltham, MA, USA), and nec1 from Biomol (Hamburg, Germany). Flag-TWEAK was produced in HEK293 cells as described elsewhere^27^. Antibodies specific for caspase-3, caspase-9, phospho-IRF-3, phospho-RIP, Fn14, TRAF1, and FLIP were obtained from Cell Signaling (Beverly, MA, USA). Caspase-8- and cIAP1-specific antibodies were purchased from Enzo Life Science (Farmingdale, USA), anti-RIPK1 and anti-PARP were provided by BD Biosciences Pharmingen (Heidelberg, Germany), and anti-tubulin was from Millipore (Schwalbach, Germany).

### Viability assay

Cells were plated (2 × 10^4^ of HeLa-RIPK3 cells or 3 × 10^4^ of HaCaT cells per well) in 96-well tissue cultures plates in 100 μl cell culture medium. Next day, cells were challenged overnight in triplicates with the reagents of interest. Cell viability was assessed by crystal violet staining. To normalize cell viability values, each plate included a triplicate of untreated cells considered as 100% viable and a triplicate of cells incubated with a cytotoxic mixture (200 ng/ml TNF, 200 ng/ml CD95L, 200 ng/ml TRAIL, 5 µg/ml CHX, 1% (w/v) sodium azide) causing maximal cell death to deliver the value for 0% viability. All other viability values were normalized according to the averages of these triplicates and analysed by the Graph Pad Prism 5 software (La Jolla, CA, USA).

### IL8 ELISA assay

Cells were cultivated (2 × 10^4^ of HeLa-RIPK3 cells or 3 × 10^4^ of HaCaT cells per well) in 96-well tissue culture plates. On the next day, medium was replaced to decrease the background of constitutive cytokine production, and cells were stimulated overnight with the indicated reagent(s). The supernatants were evaluated for production of IL8 using the human IL8 ELISA (enzyme-linked immunosorbent assay) kit BD Biosciences (Heidelberg, Germany) according to the instructions of the supplier.

### Western blot analysis

HeLa-RIPK3 variants and HaCaT cells (1 × 10^6^ cells/well) were cultivated in six-well plates and were stimulated the next day with the reagents of interest for 6 h. Adherent and, when present, detached cells were collected by scrapping with a rubber policeman and centrifugation (2 min, full speed, Eppendorf centrifuge 5417C). After two washes with ice-cold phosphate-buffered saline (PBS), the cell pellet was suspended and dissolved in 4 × Laemmli sample buffer (8 % (w/v) SDS, 0.1 M dithiothreitol, 40 % (v/v) glycerol, 0.2 M Tris, pH 8.0) supplemented with phosphatase inhibitor cocktail II (Sigma) by sonification (20 pulses) and heating for 5 min at 95 °C. After removal of the remaining insoluble debris by centrifugation (2 min, full speed, Eppendorf centrifuge 5417C) 8 µl of the lysate were applied to SDS-polyacrylamide gel electrophoresis. The segregated proteins were blotted from the gel to a nitrocellulose membrane and remaining free binding sites on the membrane were blocked by incubation for 1 h in 5% (w/v) dry milk in Tris-buffered saline with 0.1% (v/v) Tween 20. Detection of the proteins of interest were achieved with appropriate primary antibodies and horseradish peroxidase-conjugated secondary antibodies (Dako, Glostrup, Denmark and Cell Signaling Technology, Beverly, MA, USA). Nitrocellulose membrane-associated antigen–antibody complexes were visualized with the ECL Western Blotting detection reagents and analysis system Thermo Fisher Scientific (Darmstadt, Germany).

### Immunoprecipitation

HeLa-RIPK3 (5 × 10^6^) and HaCaT were used for each group. Cells were incubated with 2 µg of Fc-Flag-TWEAK^27^ for 10 or 30 min (at 37 °C and 5% CO_2_) or remained untreated as a control. After that, cells were washed four times with ice-cold PBS to stop receptor complex formation. Cells were lysed on ice by mixing with 2 ml of lysis buffer (30 mM Tris-HCl, pH 7.5, 120 mM NaCl, 50 mM β-glycerophosphate, 20 mM sodium pyrophosphate, 1 mM sodium orthovanadate, 10% glycerol, 1% Triton X-100, protease inhibitor mixture (Roche Molecular Diagnostics). Lysates were centrifuged twice (4 min, 1200 r.p.m., 4 °C) followed by another centrifugation for 30 min to remove cellular debris. A minor fraction of the resulting clear lysates was used to control for the input of the respective proteins. Lysates of untreated control cells were supplemented with 5 ng Fc-Flag-TWEAK. Receptor complexes were precipitated from the lysates by co-incubation with 40 µl of protein G beads (Roche Applied Science) overnight on a shaker at 4 °C. The beads were washed with 2 ml of lysis buffer four times by centrifugation (30 s, 5000 r.p.m., 4 °C). Finally, the pellets were mixed with lysis buffer and 4 × sample buffer and were heated at 85 °C for 10 min. After removal of the remaining insoluble debris by centrifugation (2 min, 1200 r.p.m.), the indicated proteins were detected by western blotting.

## Electronic supplementary material

supplemental material (figure S1)
