# Supplementary material for: Soluble TNF-like weak inducer of apoptosis (TWEAK) enhances poly(I:C)-induced RIPK1-mediated necroptosis
Source: Cell Death Dis. 2018 Oct 22;9(11):1084. doi: 10.1038/s41419-018-1137-1 (PMC6197222; doi:10.1038/s41419-018-1137-1)
Supplement: Supplementary file 1 — supplemental material (figure S1) [file 41419_2018_1137_MOESM1_ESM.pdf]

Supplemental data

Mohamed A. Anany et al

Soluble TNF-like weak inducer of apoptosis (TWEAK) enhances poly(I:C)-induced RIPK1-mediated necroptosis

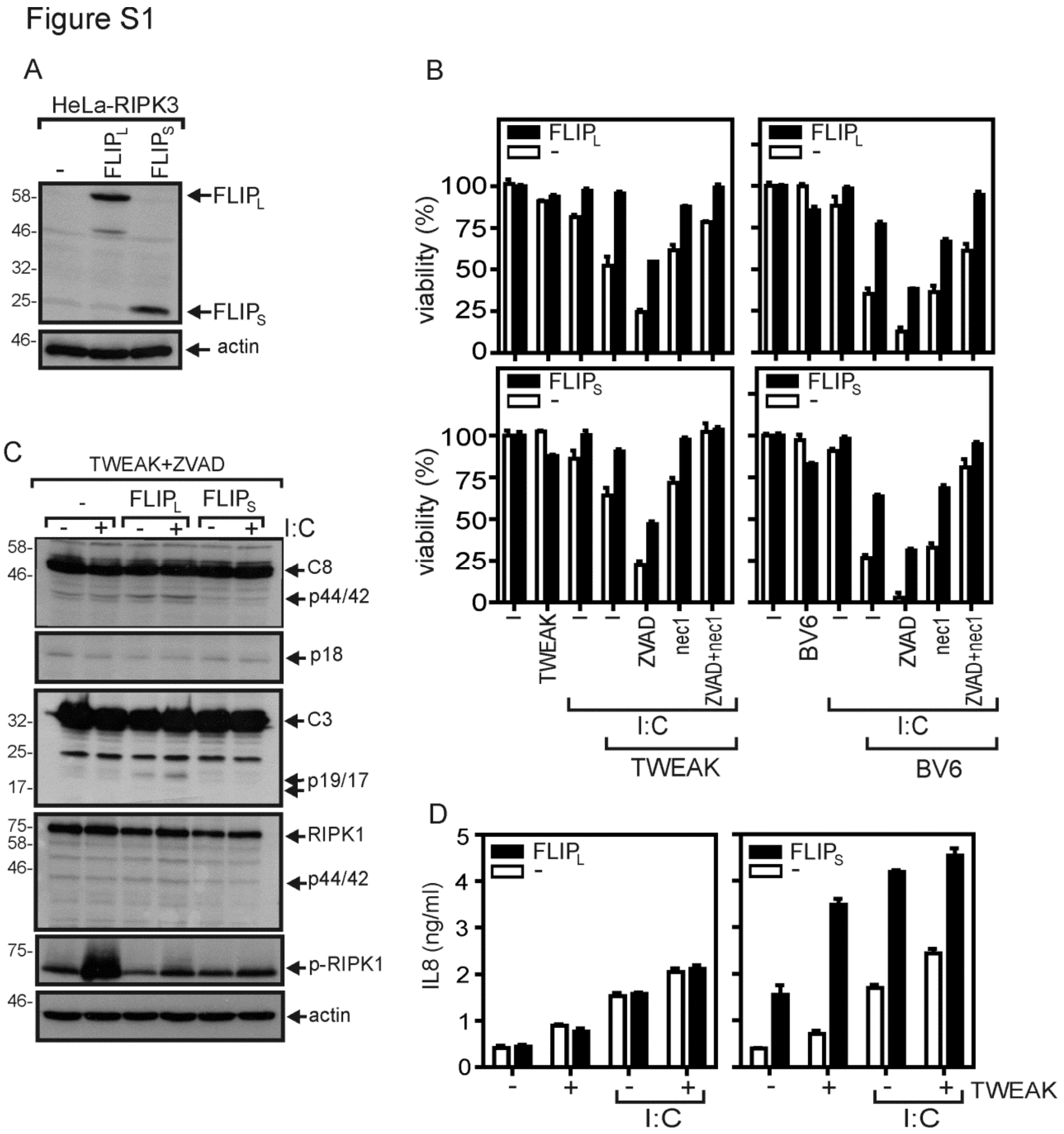

Fig. S1. a HeLa-RIPK3 (-), HeLa-RIPK3-$FLIP_L$ and HeLa-RIPK3-$FLIP_S$ were analyzed by Western blot for $FLIP_L$ and $FLIP_S$ expression. **b** The various HeLa-RIPK3 variants were stimulated with the indicated mixtures of poly(I:C) (40 µM), Flag-TWEAK (200 ng/ml), BV6 (10 µM), nec1 (90 µM) and ZVAD (20 µM) overnight. Cellular viability was finally determined by crystal violet. **c** Total cell lysates of cells which have been challenged in the presence of Flag-TWEAK (200 ng/ml) and ZVAD (20 µM) for 6 hours with poly(I:C) (40 µg/ml) were analyzed by Western Blotting. **d** Cells were stimulated with 40 µM poly(I:C) in the presence and absence of Flag-TWEAK (200 ng/ml). Next day, supernatants were analyzed using an IL8 ELISA.
